# Microglial Dysfunction and Amyloid-Beta Pathology in Alzheimer’s Disease and HIV-Associated Neurocognitive Disorders

**DOI:** 10.3390/ijms26189069

**Published:** 2025-09-17

**Authors:** George Chigozie Njoku, Georgette Djuidje Kanmogne

**Affiliations:** 1 Department of Anesthesiology, College of Medicine, University of Nebraska Medical Center, Omaha, NE 68198-4455, USA; gnjoku@unmc.edu; 2 Department of Cellular and Integrative Physiology, College of Medicine, University of Nebraska Medical Center, Omaha, NE 68198-4455, USA

**Keywords:** microglial dysfunction, amyloid-beta clearance, Alzheimer’s disease, HAND, HIV-1 Tat, scavenger receptors, neuroinflammation, phagocytosis impairment, endolysosomal dysfunction, TREM2

## Abstract

Chronic neuroinflammation and impaired protein clearance are hallmarks of neurodegenerative diseases such as Alzheimer’s disease (AD) and HIV-associated neurocognitive disorders (HAND). Central to these processes are microglia, the brain’s resident immune cells, which normally maintain brain homeostasis by clearing amyloid-beta (Aβ) and other misfolded proteins through phagocytosis and receptor-mediated degradation. However, in both AD and HAND, microglial dysfunction promotes ongoing inflammation, impaired Aβ clearance, and progressive neuronal damage. This review synthesizes evidence from human and animal studies showing how key microglial pattern recognition receptors, including the Triggering receptor expressed on myeloid cells 2 (TREM2), Toll-like receptors (TLRs), and scavenger receptors (SR-AI/II, CD36, SR-BI, CD163), coordinate Aβ sensing, uptake, and inflammatory responses. We describe how HIV infection and viral proteins such as the trans-activator of transcription (Tat) and glycoprotein 120 (gp120) disrupt these pathways by altering receptor expression, lysosomal function, and microglial metabolism, creating a cycle of neurotoxicity and amyloid buildup. We further highlight current scientific gaps in elucidating how HIV affects microglial function and implications for HAND.

## 1. Introduction

The global burden of neurodegenerative diseases has risen dramatically over the past decades, with dementia alone accounting for approximately 32.6 million disability-adjusted life years in adults aged 65 and above in 2021, while Parkinson’s disease contributed around 7.5 million disability-adjusted life years, making them among the most disabling neurological conditions worldwide [1,2,3]. A central driver of this disease burden is chronic neuroinflammation, which has emerged as a unifying pathological hallmark across neurodegenerative disorders. Within this context, microglia, the resident immune cells of the central nervous system (CNS), play a pivotal role in orchestrating inflammatory responses and maintaining homeostasis [4]. Derived from erythromyeloid progenitors in the embryonic yolk sac [5], microglia exhibit a branched, ramified morphology suited to their surveillance function [6,7]. First identified in 1919 by Spanish neuroscientist Pío del Río-Hortega as distinct from astrocytes and oligodendrocytes, microglia were originally characterized by their “resting” and “activated” states, highlighting their role in phagocytosis [8]. While initially recognized for their debris-clearing capabilities, microglia are now known to play broader roles in immune signaling, neuroprotection, myelination, and synaptic remodeling, making them essential for both brain homeostasis and defense [9,10]. In response to pathological stimuli such as infection, trauma, or neurodegeneration, they rapidly alter their gene expression, adopt an amoeboid shape, and secrete pro-inflammatory cytokines to mount an immune response [11,12]. While this acute activation is protective, sustained neuroinflammation disrupts microglial homeostasis, impairs phagocytic function, and promotes neuronal damage, contributing to the progression of neurodegenerative diseases [13].

In neurodegenerative diseases such as Alzheimer’s disease (AD) and Human Immunodeficiency Virus (HIV)-associated neurocognitive disorders (HAND), microglial dysfunction and chronic inflammation are not just passive consequences but active drivers of impaired amyloid-beta (Aβ) clearance and progressive neuronal damage [14,15,16,17]. In AD, defective microglial clearance of Aβ promotes plaque accumulation and sustained neuroinflammation [12]. In HAND, HIV infection drives persistent microglial activation and disrupts homeostatic functions, creating a neuroinflammatory environment that worsens neuronal injury [14]. It is still unclear whether the extent of neurodegeneration and neuronal death is directly tied to the loss of microglial homeostasis. However, growing evidence suggests that HIV-induced neuroinflammation disrupts microglial function, contributing to impaired immune surveillance, reduced clearance of neurotoxic proteins such as Aβ, and increased neuronal vulnerability [14,15,16,17]. Hence, this review aims to explore the mechanisms by which microglial dysfunction contributes to impaired Aβ clearance and the implication for HAND. We will discuss the effects of HIV and viral proteins on microglia activation and function, including its phagocytic and Aβ clearance function, and identify the key knowledge gaps that remain to be addressed through future research.

## 2. Microglia: History, Function, and Role in CNS Homeostasis

### 2.1. Microglia Discovery and Historical Perspective

The concept of immune surveillance within the brain can be traced back more than a century to the foundational work of Ilya Méchnikov, who introduced the idea of phagocytosis after observing mobile, engulfing cells in starfish larvae [18]. Years later, Nicolás Achúcarro identified similar cells in the brains of rabbits infected with the rabies virus, noting their role in clearing damaged neurons [19]. Subsequently, after refining the staining techniques initially developed by Santiago Ramón y Cajal [20], Del Río-Hortega successfully identified and characterized microglia as a distinct glial population within the CNS, specialized in response to injury and infection [8,19,21]. Del Río-Hortega’s innovations enabled detailed cellular visualization and established microglia as non-neuroectodermal, yolk-sac-derived immune sentinels of the brain. These early discoveries laid the groundwork for our understanding of microglial identity and surveillance functions, processes now recognized as central to the pathophysiology of neurodegenerative diseases [22].

### 2.2. Homeostatic Functions and Dynamic Surveillance

Unlike other CNS cells derived from neuroectodermal progenitors, microglia originate from yolk sac progenitors during early embryogenesis. They constitute approximately 5–10% of the total cell population in the human brain [6,7] and, in addition to endothelial cells, represent the only brain-resident cell type of non-neuroectodermal origin [5]. Microglia migrate into the developing brain prior to the formation of the blood–brain barrier (BBB), colonizing the neuroepithelium where they proliferate, spread throughout the CNS, and differentiate into mature, homeostatic microglia [5,23,24].

In the adult brain, microglia are highly dynamic, constantly extending and retracting their processes to monitor the surrounding parenchyma and maintain tissue integrity [25,26]. They achieve this surveillance through a diverse array of pattern recognition receptors (PRRs), including Toll-like receptors (TLRs) such as TLR4 and TLR1/2, as well as co-receptors like cluster of differentiation (CD)14, CD36, and CD47, which allow microglia to sense pathogen-associated molecular patterns (PAMPs) and damage-associated molecular patterns (DAMPs) [25,26]. Although the exact mechanisms regulating microglia motility and morphological changes remain incompletely understood, studies suggest that purinergic receptors, ion channels, and neurotransmitters play important regulatory roles in this process [11,27,28,29]. Microglia also express chemokine receptors such as CX3CR1 and CXCR4, alongside integrins such as CD11b and CD11c, which contribute to their migration and enhance their ability to engage and phagocytose target cells [30,31,32]. CD11b is constitutively expressed, whereas CD11c is typically upregulated during microglial activation [30,33,34]. Common molecular markers of homeostatic microglia include hexosaminidase subunit beta, P2Y purinoceptor 12, S100 calcium-binding proteins A8 and A9, ionized calcium-binding adapter molecule 1, transmembrane protein 119, G protein-coupled receptor 34, sialic acid-binding Ig-like lectin H, the triggering receptor expressed on myeloid cells 2 (TREM2), and olfactomedin-like protein 3, which help distinguish them from other CNS cell types and from disease-associated states [35,36]. In addition to their immune surveillance roles, homeostatic microglia also contribute to synaptic pruning, neurotrophic support, and myelination, thereby preserving CNS structure and function under physiological conditions [37,38,39,40]. Altogether, these features show how microglia are uniquely suited to monitor and support brain function under normal conditions. Loss or impairment of this microglial regulatory role can contribute to reduced clearance of harmful proteins and increased inflammation seen in neurodegenerative diseases such as AD and HAND [41].

### 2.3. Specialized Immune Functions and Transition to Neuroinflammation

In response to environmental perturbations, microglia rapidly transition from a homeostatic, ramified phenotype to an activated, amoeboid form [42,43]. This phenotypic switch can be triggered by PAMPs such as viral nucleic acids, bacterial deoxyribonucleic acid (DNA), and lipopolysaccharide (LPS) [44,45], as well as endogenous DAMPs, including heat shock proteins, high-mobility group box 1 protein, and mitochondrial DNA [46]. Recognition of these molecules by the immune system triggers microglial activation [47,48]. Activated microglia upregulate CD11c and secrete pro-inflammatory mediators including tumor necrosis factor-alpha (TNF-α), interleukin (IL)-1β, IL-6, nitric oxide (NO), and reactive oxygen species (ROS), leading to synaptic dysfunction, neuronal injury, and death [13,42,49]. Significantly, activated microglia also suppress anti-inflammatory transforming growth factor-beta via the nuclear factor kappa-light-chain-enhancer of activated B cells (NF-κB)-mediated signaling [50,51], thereby disrupting immune homeostasis, promoting CNS tissue damage, and behavioral impairments [50].

Although transient microglial activation may serve a protective function during acute insults, prolonged or dysregulated activation results in chronic neuroinflammation, which is a hallmark of neurodegenerative diseases such as AD and HAND. Notably, one key consequence of prolonged microglial activation is dysregulation of glutaminase activity, which leads to excessive production of glutamate [52]; glutamate accumulates in the extracellular space where it overstimulates *N*-methyl-d-aspartate receptors, particularly the extrasynaptic subtype [52,53], resulting in neuronal calcium (Ca^2+^) overload and excitotoxic cascades that compromise cellular integrity [52,53]. Beyond excitotoxicity, Ca^2+^ plays a dual pathological role in amyloidogenesis: intracellular Ca^2+^ activates signaling pathways that upregulate β-site APP-cleaving enzyme 1 and γ-secretase, driving amyloidogenic APP processing and increased production of Aβ_42_ peptides [54]; at the same time, Ca^2+^ directly modulates amyloid fibril formation. Experimental evidence shows that Ca^2+^ binding can reshape aggregation pathways, as in the case of S100 calcium-binding protein A9 forming “worm-like” fibrils with distinct mechanical properties [55], while variations in ionic strength and protein concentration critically determine fibril polymorphism, as demonstrated for α-synuclein aggregation [56]. These processes underscore how Ca^2+^ overload not only initiates Aβ production but also accelerates fibrillar growth under permissive cellular conditions. The accumulation of Aβ further amplifies neuronal dysfunction and loss, serving as a central hallmark of both AD and HAND pathogenesis [57,58,59].

## 3. Microglial Role in Aβ Clearance

AD is the leading cause of dementia worldwide and significantly impacts quality of life [60,61]. Currently, there is no cure, and available treatments focus on alleviating symptoms, slowing disease progression, and managing the risks associated with cognitive decline [62,63]. Numerous hypotheses have been proposed to explain the causes of AD, which include neuroinflammation [64,65], mitochondrial dysfunction [66,67,68,69], calcium dysregulation [70], and impaired autophagy [71,72].

In AD, a hallmark of disease pathology is the extracellular deposition of Aβ, which is toxic to neurons [73], drives chronic inflammation [74], aggregates, and forms senile plaques that disrupt cellular function and which are linked to cognitive impairments [61]. Another hallmark of AD pathology is hyperphosphorylation of tau proteins, resulting in neurofibrillary tangles (NFTs) that are also linked to cognitive impairments [75,76]. NFTs are associated with the late stage of AD (LOAD), when the symptoms are generally irreversible [77], whereas Aβ plaque accumulation occurs at earlier stages of AD (EOAD), before the manifestation of cognitive deficits [77]. Notably, Aβ plaques are also detected in LOAD [13,78]. NFTs and Aβ aggregates cause neuronal damage, synaptic dysfunction, and cognitive decline [13,79].

One of the brain’s key defenders in AD is microglia [64,80]. Under normal conditions, Aβ is primarily produced by neurons through enzymatic cleavage of APP, while its clearance is largely mediated by microglia via mechanisms such as phagocytosis, enzymatic degradation, and transport across the BBB [77,81,82,83]. However, in the context of AD, this balance becomes disrupted. As the disease progresses, microglia respond to accumulating Aβ by increased proliferation and activation [13,84], releasing inflammatory cytokines and ROS [13,85,86]. Instead of repairing the damages, this prolonged inflammatory state further impairs microglial function, diminishing microglia’s capacity to clear Aβ. This leads to greater accumulation of Aβ and plaques, progression of pathology, and worsening neurocognitive decline [12]. Yet despite their increased numbers, microglia capacity to clear Aβ diminishes significantly [87,88]. This raises critical questions: how much Aβ can microglia effectively clear before reaching their functional limit? At what point does their protective role shift toward a pathological one, ultimately contributing to further Aβ accumulation? These questions remain unanswered but are essential to understanding microglial dysfunction and failure of its clearance function in AD and other neurodegenerative diseases. Supporting evidence suggests that as Aβ levels rise, microglia become overstimulated and enter a chronic inflammatory state that paradoxically impairs their phagocytic function [89,90,91,92,93]. Thus, while microglia initially act as defenders, there comes a tipping point where their response exacerbates the pathology.

### 3.1. Phagocytosis and Degradation of Aβ

Phagocytosis is an essential cellular process that maintains tissue homeostasis and regulates immune responses [94]. It relies on phagocytes receptors to recognize specific cells or proteins as targets for engulfment. Beyond this receptor-mediated recognition, the complement system plays a key role by tagging (opsonizing) pathogens or protein aggregates, making them more easily identified and cleared by phagocytic cells [94,95]. In AD, microglial cells recognize Aβ through several receptors, including TREM2, TLRs, low-density lipoprotein receptor-related-1 (LRP1), the receptor for advanced glycation end products (RAGE), and scavenger receptors (SCARA1, CD36, CD163, and SCARB-1) [13,96,97]. Once Aβ binds to these receptors, intracellular signaling pathways are activated, triggering cytoskeletal rearrangements that enable microglia to engulf Aβ through endocytosis. After internalization, Aβ is enclosed within endocytic vesicles and transported to lysosomes, where it is degraded by proteolytic enzymes such as cathepsins and insulin-degrading enzyme [98,99]. This degradation process prevents the extracellular accumulation of Aβ, thereby reducing Aβ-induced inflammation and synaptic dysfunction.

### 3.2. Microglia Surface Receptors and Their Response to Aβ

#### 3.2.1. TREM2

##### Overview of TREM2 in the CNS

TREM2 is a transmembrane immunoglobulin-like receptor expressed predominantly on myeloid-lineage cells, including dendritic cells, tissue macrophages, and microglia, which are the principal innate immune cells in the CNS [40]. TREM2 is essential for maintaining brain homeostasis and regulating key microglial functions, including chemotaxis, survival, proliferation, and phagocytosis [100]. Although the precise repertoire of endogenous TREM2 ligands remains incompletely defined, available evidence suggests it can recognize oxidized lipids, lipoproteins, and molecular components derived from apoptotic or damaged cells [101]. Notably, the extracellular domain of TREM2 has also been shown to bind LPS, suggesting a potential role in pathogen sensing [100]. Importantly, TREM2 has been shown to binds oligomeric Aβ with high affinity, a function compromised by AD-associated mutations, such as the arginine-to-histidine substitution at position 47 (R47H)-variant [102]. Genetic studies have robustly linked the R47H mutation to a 2–4-fold increased risk of LOAD, comparable to the risk conferred by the apolipoprotein E (APOE) ε4 allele [103]. Functional studies indicate that a TREM2 deficiency impairs Aβ phagocytosis, cytokine responses, and microglial migration while also disrupting ion homeostasis, including membrane depolarization and K^+^ currents [102,103].

##### Mechanisms and Functional Significance of TREM2 in Aβ and Tau Clearance

Several lines of evidence indicate that TREM2 plays a critical role in modulating microglial interactions with Aβ and tau in AD pathology [11,100,104,105]. Overexpression of TREM2 reduces microglial proinflammatory signaling, whereas its deletion or knockdown enhances microglia activation and inflammation and drives a neurodegenerative microglial phenotype [11,100]. Beyond its effects on inflammation and tau phosphorylation, recent studies have shown that TREM2 also facilitates microglial recognition and containment of Aβ plaques by promoting microglial clustering and limiting the local spread of phosphorylated tau [106].

Mechanistically, TREM2 recognizes lipid and lipoprotein ligands, such as APOE and clusterin, as well as phospholipids exposed on damaged neurons and Aβ aggregates [100,107]. Upon ligand binding, TREM2 interacts with its adaptor protein, the DNAX-activating protein of 12 kDa (also known as the TYRO protein tyrosine kinase binding protein), which contains immunoreceptor tyrosine-based activation motifs. Phosphorylation of the DNAX-activating protein of 12 kilodaltons immunoreceptor tyrosine-based activation motifs recruits spleen tyrosine kinase, triggering downstream phosphoinositide 3-kinase (PI3K)/protein kinase B (AKT) and extracellular signal-regulated kinase 1/2 (ERK1/2) pathways that drive cytoskeletal rearrangements required for efficient Aβ engulfment [108]. PI3K/AKT activation further inhibits glycogen synthase kinase-3 beta, which reduces tau hyperphosphorylation and limits the local propagation of tau pathology [109,110]. In parallel, TREM2 enhances microglial lipid metabolism and cholesterol efflux, boosting microglial capacity to clear lipid-rich Aβ plaques [111,112]. By promoting microglial clustering, TREM2 also helps form a physical barrier around plaques, containing toxic species and protecting surrounding neurons [11].

The functional significance of these mechanisms is evident in vivo. The loss of TREM2 impairs microglial recruitment and clustering, reduces phagocytic capacity, and shifts microglia toward a more pro-inflammatory, neurodegenerative state [113]. In TREM2-deficient presenilin-2 amyloid precursor protein (PS2APP) mice, microglia–plaque connections are impaired, leading to more diffuse plaque structures, elevated levels of toxic Aβ, reduced microglial proliferation, and increased neuronal dystrophy [114]. Moreover, TREM2 deficiency promotes the accumulation of pathological tau in the presence of Aβ [115,116]. In such cases, the amount of phosphorylated tau protein near plaques increases significantly, exacerbating tau aggregation and spreading, and ultimately contributing to cortical atrophy [115,116]. In contrast, in the absence of Aβ pathology, TREM2 loss does not appear to influence tau burden [106,115]. These findings suggest that TREM2 may mitigate tau-driven neurodegeneration by limiting Aβ-induced propagation of pathological tau, thereby slowing the progression of AD [106,115].

Furthermore, in vivo studies using 5XFAD transgenic mice lacking TREM2 have revealed markedly increased Aβ deposition and neurodegeneration in the hippocampus, particularly by 8.5 months of age, whereas the cortex appears relatively unaffected [117]. This was accompanied by a significant accumulation of insoluble Aβ_40_ and Aβ_42_ [117]. However, at earlier stages (4 months), TREM2 deficiency did not significantly affect the Aβ burden, suggesting that the impact of TREM2 loss depends on disease progression and the specific brain region involved [117]. Supporting this idea of stage- and region-specific effects, results from other AD mouse models show similar patterns. In APPPS1-21 mice, TREM2 deficiency led to reduced microglial clustering around plaques and different effects on Aβ levels depending on brain region and age [118]. At four months, cortical Aβ levels remained unchanged, while hippocampal Aβ levels decreased [118]; by eight months, cortical Aβ burden increased, while hippocampal levels showed no further change [119]. These findings indicate that certain brain regions, such as the hippocampus, may be more vulnerable to Aβ accumulation and neurodegeneration when TREM2 is lost, while others, like the cortex, may be less affected during the same disease stages. Conversely, enhanced TREM2 expression achieved through transgenic overexpression of human TREM2 in 5XFAD mice reduced Aβ plaque burden and modified microglial transcriptomic signatures, supporting a dose-dependent protective role for TREM2 [120]. Notably, multiple studies have demonstrated that TREM2 plays a role in AD pathogenesis by regulating microglial phagocytosis, inflammatory response, proliferation, and lipid metabolism [40,111,112,121,122,123,124]. This highlights the crosstalk between TREM2, microglia, and AD pathology and TREM2’s critical role in coordinating microglial responses to both Aβ and tau.

##### TREM2 Regulates Microglial Metabolism

TREM2 supports microglial metabolic fitness by regulating key pathways that control energy balance, including the mammalian target of the rapamycin (mTOR) pathway and glycolysis [125]. mTOR, particularly mTOR complex 1 (mTORC1), is a nutrient-sensing kinase that promotes glycolysis, mitochondrial biogenesis, and protein synthesis and suppresses excessive autophagy to maintain energy balance [126]. In microglia, active mTORC1 is essential for providing the energy and biosynthetic capacity needed for phagocytosis and Aβ degradation [127]. In TREM2-deficient microglia, mTORC1 activity is suppressed while adenosine monophosphate-activated protein kinase phosphorylation is elevated, indicating a shift toward an energy-deficient, catabolic state [128]. Reduced mTORC1 signaling impairs glycolytic flux and mitochondrial function, leading to lower adenosine triphosphate levels, decreased mitochondrial mass, and accumulation of autophagic vesicles [128].

Selective deletion of tuberous sclerosis complex 1 (TSC1), a negative regulator of mTORC1, in microglia reactivates mTORC1 signaling, upregulates TREM2 expression, and enhances Aβ clearance in 5XFAD mice [125]. These changes are associated with reduced synaptic spine loss and improved cognitive performance. Notably, combined deletion of TSC1 and TREM2 abolishes these benefits, demonstrating that TREM2 acts downstream of mTORC1 in coordinating microglial Aβ clearance [125]. In addition, TSC1-deficient microglia show increased expression of lysosomal markers CD68 and lysosome-associated membrane glycoprotein 1, greater lysosomal activity, and enhanced Aβ uptake and degradation [125]. These effects are reversed by rapamycin treatment, both in vitro and in vivo. Chronic inhibition of mTORC1 with rapamycin lowers TREM2 expression, impairs microglial function, and increases Aβ plaque burden [125].

#### 3.2.2. Toll-like Receptors

TLRs are a major family of the PRRs found on cell membranes that are integral to immune response, including in the CNS; they are recognized for their ability to detect both endogenous and exogenous signals of infection or cellular damage [129,130]. In humans and other mammals, there are 13 structurally different TLRs that recognize conserved viral, bacterial, and fungal particles expressed in various cell types, including B cells, T cells, macrophages, monocytes, dendritic cells, glial cells (microglia, oligodendrocytes, and astrocytes), and neurons [131,132]. These TLRs have been widely studied for their roles in regulating neuroinflammation and Aβ clearance in AD [130]. Depending on the specific TLR subtype activated, disease stage, and surrounding cellular environment, TLR signaling can have either protective or detrimental effects on AD pathology [130,133]. For example, some TLRs promote microglial phagocytosis and clearance of Aβ, whereas others may exacerbate chronic inflammation and contribute to neurotoxicity.

##### Pathological Relevance

In AD, microglial TLRs are closely associated with Aβ plaques and act as key sensors of misfolded protein aggregates and damage signals [86]. TLR2 and TLR4 are consistently upregulated in microglia localized around Aβ plaques in both human postmortem tissue and transgenic mouse models, indicating their active engagement in situ [11,134]. TLR4 recognizes fibrillar Aβ in cooperation with co-receptors CD14 and myeloid differentiation factor 2 (MD-2), initiating robust proinflammatory signaling cascades [135,136]. Similarly, TLR2 colocalizes with CD14 and is enriched in plaque-associated microglia, supporting its role in innate recognition of Aβ [135,136]. In contrast, TLR9, typically localized in endosomes, does not show major upregulation near plaques but has been implicated in peripheral immune responses that impact AD pathology in the CNS [137].

##### Evidence from Human and Animal Models

Data from both human AD brains and animal models reinforce the pathological relevance of TLR-mediated microglial dysfunction (Table 1). In postmortem brain tissues, TLR2 and TLR4 immunoreactivity is elevated in microglia adjacent to amyloid plaques [138], while APP23 transgenic mice showed significant transcriptional upregulation of TLR2, TLR4, TLR5, TLR7, and TLR9 in plaque-bearing regions [139]. Functional studies in knockout animals reveal divergent roles: TLR4^−/−^ mice exhibit impaired microglial Aβ uptake, increased amyloid plaque burden, and worsened cognitive performance [140,141]. In contrast, TLR2^−/−^ mice demonstrate enhanced microglial phagocytosis and a marked reduction in proinflammatory cytokine expression, including TNF-α, IL-1β, IL-6, and iNOS, as well as lower expression of integrin markers such as CD11a, CD11b, and CD68, suggesting that TLR2 activation skews microglia toward a proinflammatory, less phagocytic state [142,143]. In vitro studies have demonstrated that stimulation of TLR2 on microglia with peptidoglycan enhances the internalization of AD-associated Aβ-peptide, a process that is mediated in part by formyl peptide receptor 2 [144]. However, this effect is considered context-dependent, as it was observed under specific experimental conditions and may not fully translate to the in vivo brain microenvironment, where additional factors such as disease stage, cellular state, and interactions with other receptors can modulate microglial Aβ uptake. Neutralizing antibodies against CD14, TLR2, or TLR4 reduce Aβ binding and ROS production in mouse microglia and human monocytes, which further confirmed the involvement of these receptors in amyloid sensing and binding [145]. TLR9, which is primarily localized in endosomes, plays a relatively minor role in CNS-resident microglia but can exert significant effects through peripheral immune activation. Systemic administration of cytosine–phosphate–guanosine oligodeoxynucleotides (CpG ODNs), synthetic unmethylated DNA sequences that specifically activate TLR9, has been shown to boost peripheral innate immunity and promote the recruitment of monocyte-derived macrophages into the brain [146]. In transgenic AD mice, this increased infiltration of peripheral macrophages enhanced Aβ clearance, resulting in a 66% reduction in cortical amyloid burden and improved spatial memory [146].

#### 3.2.3. Scavenger Receptors (SRs)

SRs are multifunctional PRRs that bind a broad array of ligands, including misfolded proteins, microbial components, and modified self-antigens [148,149]. In AD, microglia utilize several SRs for the recognition, uptake, and degradation of Aβ, with distinct receptors engaging in either protective clearance or triggering inflammatory signaling that can exacerbate neuroinflammation [150,151]. Class A and Class B SRs have received particular attention due to their well-documented roles in Aβ recognition, uptake, and clearance, as well as their impact on disease progression [148]. Their roles extend beyond Aβ binding to include the modulation of intracellular signaling cascades, glial activation states, and crosstalk with innate immune receptors such as TLRs. These roles are summarized in Table 2.

##### Class A Scavenger Receptors (SR-AI/II)

SR-AI is a trimer composed of three identical subunits, each containing a short intracellular region, a single transmembrane segment, and several specialized extracellular domains, including an α-helical coiled-coil domain, a collagen-like region, and a cysteine-rich domain at the C-terminus [152]. Class A SRs, including SR-AI/II (also known as SCARA1), are predominantly expressed in microglia and, to a lesser extent, astrocytes [149,153]. These receptors play a role in the phagocytic uptake and lysosomal degradation of Aβ, particularly fibrillar and oligomeric Aβ. Upon binding to Aβ, SR-A mediates its internalization, likely via clathrin-independent macropinocytosis, and delivers internalized Aβ to lysosomal compartments [154]. Concurrently, SR-A also activates the PI3K/NF-κB pathway as well as MAPK pathways, including c-Jun N-terminal kinase (JNK) and p38 MAPK, to promote actin remodeling and microglial migration toward Aβ deposits [155]. SR-A activation has been shown to suppress TLR4 signaling, suggesting it may help limit excessive neuroinflammation, particularly during early stages of disease when pro-inflammatory responses are first triggered [156]. Studies using the synthetic SR-A ligand XD4 demonstrated that SR-A activation enhances microglial Aβ uptake while simultaneously dampening the secretion of TNF-α and IL-1β, suggesting a dual function in Aβ clearance and modulation of inflammatory responses [151].

Evidence from studies in animal models underscores the importance of SR-A in early-stage AD pathology. In PS1/APP transgenic mice, genetic deletion of SR-AI leads to significantly impaired microglia Aβ uptake, accompanied by accelerated plaque deposition and increased mortality [155]. SR-AI-deficient mice exhibit a marked reduction in microglia uptake of soluble Aβ and a 60% increase in Aβ accumulation in the brain parenchyma [155]. Similarly, SR-A deficiency disrupts microglial migration and phagocytosis, further exacerbating Aβ pathology [157]. The protective role of SR-A in supporting microglial migration and Aβ phagocytosis helps limit plaque accumulation during the early stages of AD; however, its expression declines markedly as the disease progresses, which may contribute to reduced Aβ clearance in later stages [157,158,159,160]. This SR-A decline correlates with increased levels of inflammatory cytokines and a shift toward a dysfunctional, proinflammatory microglial phenotype [161]. Postmortem analyses of human AD brains also showed that SR-A expression in early-stage microglia was robust and showed significantly reduced expression in regions of extensive plaque burden and inflammation [161]. These findings collectively suggest that SR-A contributes to Aβ homeostasis during early disease phases but becomes compromised under sustained inflammatory stress, thereby participating in the progression of microglial dysfunction.

##### Class B Scavenger Receptors (CD36 and SR-BI)

Class B SRs, including CD36 (SCARB-2) and SR-BI (SCARB-1), are defined by their membrane-spanning N- and C-terminus and a central extracellular loop [162]. These receptors are involved in lipid metabolism, pathogen recognition, and the clearance of modified self-antigens [163,164]. In AD, they have emerged as key regulators of Aβ-induced microglial activation and neurotoxicity [165]. CD36 was initially identified as a receptor for thrombospondin and later for parasitized erythrocytes, oxidized LDL, β-glucans, and advanced glycation end-product–modified proteins [153,166]. Due to its promiscuous ligand binding capacity, CD36 is at the intersection of metabolic dysfunction and innate immune activation in neurodegeneration.

Among Class B SRs, CD36 is the most extensively studied in the context of AD and serves as a central mediator of both Aβ clearance and innate immune activation [167]. CD36 is expressed on microglia, macrophages, and cerebrovascular endothelial cells, and it binds fibrillar Aβ with high affinity [168]. Upon ligand binding, CD36 recruits the proto-oncogene tyrosine-protein kinase Lyn, a member of the sarcoma family of tyrosine kinases, to its cytoplasmic tail, initiating the assembly of a heterotrimeric complex with TLR4 and TLR6 in lipid raft domains [169]. This receptor complex triggers a cascade of downstream signaling events involving the activation of MAPK and NF-κB pathways, resulting in the production of ROS, NO, and proinflammatory cytokines such as TNF-α and IL-1β [169,170]. Additionally, CD36 binding to Aβ primes the NOD-like receptor protein 3 (NLRP3) inflammasome, amplifying the inflammatory response and promoting neurotoxicity [171]. This dual functionality positions CD36 as a receptor that not only facilitates Aβ uptake but also transforms microglia into inflammatory effectors that may contribute to neuronal injury.

Animal studies have provided strong support for the pathogenic role of CD36 in Aβ-induced inflammation. CD36-deficient mice exhibit significantly reduced microglial recruitment and cytokine production following intracerebral Aβ injection [172]. In vitro, CD36^–/–^ microglia and macrophages display impaired ROS generation and diminished secretion of proinflammatory mediators in response to Aβ exposure [172,173]. Importantly, CD36 acts in synergy with TLR4 and TLR6. Upon Aβ binding, a heterotrimeric CD36–TLR4–TLR6 complex assembles in lipid rafts, amplifying inflammatory signaling and triggering NLRP3 inflammasome priming [174,175]. This leads to IL-1β upregulation, ROS production, and sustained microglial activation, features that contribute to neurotoxicity and disease progression [174]. While this axis enhances the microglial response to Aβ, chronic CD36 activation exacerbates tissue damage, suggesting that CD36’s role is highly context-dependent.

In human AD brains, CD36 expression is markedly increased in microglia clustered around Aβ plaques, particularly in regions of dense-core fibrillar Aβ deposition [153]; it co-localizes with Aβ in reactive microglia; and its expression strongly correlates with both plaque burden and inflammatory gene expression [176]. However, CD36 has not been detected in healthy brains from age-matched non-AD controls with no Aβ deposit [177], underscoring its pathology-dependent induction. Like SR-A, CD36 expression appears to wane in later stages of disease, likely reflecting microglial exhaustion or chronic desensitization to persistent inflammatory stimuli [169]. This progressive downregulation may impair Aβ clearance, creating a self-reinforcing loop of chronic inflammation and plaque accumulation. Collectively, findings from these studies support a model in which CD36 mediates early protective responses but later contributes to neurodegeneration through sustained inflammatory activation [178].

SR-BI (also known as SCARB1) is a Class B scavenger receptor primarily involved in selective lipid uptake and is expressed on brain endothelial cells, astrocytes, and microglia [179]. Some in vitro studies suggest that SR-BI can bind fibrillar Aβ and may facilitate its brain-to-blood efflux, potentially contributing to CNS Aβ clearance mechanisms [180,181]. While direct evidence of SR-BI-mediated Aβ transcytosis across the BBB is still limited, its involvement in vascular lipid homeostasis and glial responses suggests a modulatory role [182]. Its functional relevance in AD remains incompletely defined, although emerging data suggest that SR-BI may contribute to cerebrovascular protection during early disease stages [183]. CD163, although less studied in AD, is a hemoglobin–haptoglobin scavenger receptor typically expressed by tissue-resident macrophages with anti-inflammatory phenotypes [184,185]. Under neuroinflammatory conditions, CD163 expression is upregulated in perivascular macrophages and activated microglia [186].

**Table 2:** Scavenger receptors involved in microglial Aβ clearance and neuroinflammation in AD.

Receptor	Aβ Role	Mechanism of Action	Human Evidence	Animal Evidence	References
SR-AI/II (SCARA1/MSR1)	Aβ binding, uptake, degradation	Clathrin-independent macropinocytosis; Aβ targeting to lysosomes; activates PI3K/NF-κB, JNK, and p38 MAPK; suppresses TLR4 signaling	High expression in early-stage microglia; decreased in advanced AD with plaque burden	SR-A knockout in PS1/APP mice leads to reduced Aβ uptake, increased plaques, impaired migration	[155,156,157,160,161]
CD36 (SCARB2)	Aβ recognition, uptake, inflammation	Binds fibrillar Aβ; forms CD36–TLR4–TLR6 complex; activates MAPK, NF-κB, and NLRP3 inflammasome; promotes ROS, TNF-α, and IL-1β production	Upregulated in plaque-associated microglia; absent in non-AD controls; declines with disease progression	CD36^–/–^ mice show reduced cytokines and microglial recruitment to Aβ; reduced ROS and proinflammatory output	[167,171,172,176,177]
SR-BI (SCARB1)	Potential role in Aβ transport	Binds fibrillar Aβ; may mediate transcytosis across the BBB; limited microglial expression	Expressed on brain endothelial cells; involvement in Aβ efflux from brain to periphery hypothesized	Reduced SCARB1 leads to worsened cognitive outcomes in AD mice; no change in microglial clustering.	[168]
CD163	Unknown role in Aβ clearance; immune modulation	Hemoglobin–haptoglobin scavenger; anti-inflammatory via IL-10, HO-1 signaling; possible role in resolution of neuroinflammation	Detected in microglia of people with HAND; not well studied in AD	Upregulated in neuroinflammation; may reflect anti-inflammatory activation	[186,187]

Abbreviations: SCARA1/MSR1: scavenger receptor class A member 1/macrophage scavenger receptor 1; SCARB2: scavenger receptor class B member 2; PI3K phosphoinositide 3-kinase; JNK: c-Jun N-terminal kinase; p38 MAPK: p38 mitogen-activated protein kinase; TLR: toll-like receptor; NF-κB: nuclear factor kappa-light-chain-enhancer of activated B cells; NLRP3: NOD-, LRR-, and pyrin domain-containing protein 3; ROS: reactive oxygen species; TNF-α: tumor necrosis factor-alpha; IL: interleukin; PS1/APP: presenilin-1/amyloid precursor protein; CD36: cluster of differentiation 36; HO-1: heme oxygenase-1; HAND: HIV-associated neurocognitive disorders; AD: Alzheimer’s disease.

#### 3.2.4. Receptor for Advanced Glycation End Products (RAGE)

The RAGE has been recognized as a multiligand receptor belonging to the immunoglobulin superfamily and it is expressed in a variety of cell types, including neurons, endothelial cells, smooth muscle cells, and macrophages [188,189]. In AD, the RAGE contributes to disease pathology by facilitating the BBB transport and influx of circulating Aβ into the brain [190]. Once Aβ is transported into the brain parenchyma, its interaction with the RAGE on neurons, microglia, and endothelial cells triggers multiple downstream signaling cascades. For example, Aβ–RAGE binding activates the NF-κB pathway in neurons and microglia, promoting the production and release of pro-inflammatory cytokines that amplify neuroinflammation [191]. On brain endothelial cells, Aβ-RAGE binding further activates MAPK pathways, including JNK and ERK, which upregulate the expression of matrix metalloproteinase-2, contributing to BBB disruption and vascular inflammation [192]. Thus, by mediating Aβ transport and initiating cell-type-specific signaling events, the RAGE acts as a key link between the peripheral Aβ burden and central neuroinflammatory and vascular pathology in AD.

In microglia, the RAGE-Aβ axis activates the p38 MAPK pathway, further enhancing the production of proinflammatory cytokines. This is the case in the transgenic AD mouse model, where Aβ exposure to microglia overexpressing the RAGE led to a significant increase in IL-1β and TNF-α levels, and this correlated with increased phosphorylation of p38 MAPK and ERK1/2 [193]. These inflammatory responses likely contribute to the neuronal damage and memory deficits observed in AD. Additionally, Aβ binding to the neuronal RAGE has been shown to promote oxidative stress, further exacerbating neurodegeneration [191,194,195].

#### 3.2.5. Low-Density Lipoprotein Receptor-Protein 1 (LRP1)

LRP1 is widely expressed in CNS cells, including neurons, astrocytes, endothelial cells, and microglia, where it functions as both an endocytic receptor and a signaling mediator in response to Aβ [196]. It facilitates Aβ uptake and degradation directly or via complexes with ApoE, α2-macroglobulin, or clusterin [196], and at the BBB, it mediates Aβ efflux into the peripheral circulation [197]. Age-related decline in LRP1 expression and reduced ApoE4 binding impair Aβ clearance [198], and ApoE can also compete with Aβ for LRP1 binding, potentially affecting Aβ clearance efficiency [199]. Knockdown of LRP1 in brain-derived microglial clone-2 cells and primary astrocytes significantly reduced Aβ uptake and lysosomal degradation [200] and enhanced Aβ_42_-induced production of pro-inflammatory cytokines, including TNF-α, IL-1β, and IL-6, via activation of NF-κB and MAPK pathways [201]. This effect was accompanied by elevated expression of TLR4, MyD88, and TRAF6, which function as upstream signaling adaptors that link Aβ recognition to the activation of these inflammatory pathways [201]. Similarly, Yang et al. demonstrated that LRP1 negatively regulates microglial activation. Using LRP1 knockdown or pharmacological inhibition, they showed that loss of LRP1 in primary microglia activated JNK and NF-κB signaling pathways, leading to elevated cytokine expression [202]. Moreover, pro-inflammatory stimuli like LPS suppressed LRP1 expression, creating a feed-forward loop of inflammation. Interestingly, inhibiting NF-κB activation not only reversed cytokine upregulation but also restored LRP1 expression, suggesting that LRP1 modulates microglial behavior through reciprocal interactions with key inflammatory signaling networks [202]. Evidence from these studies has shown that inhibition of the NF-κB, p38, and JNK pathways reduces cytokine production, suggesting that LRP1 plays a suppressive role in glial-mediated inflammation.

## 4. Microglial Activation and Dysfunction in HIV Infection

HIV enters the CNS early in infection, establishing long-lived reservoirs in brain-resident macrophages and microglia [203]. Several HIV proteins, including trans-activator of transcription (Tat), glycoprotein 120 (gp120), viral protein R (Vpr), Viral protein U (Vpu) and negative regulatory factor (Nef) are chronically expressed and released from infected or activated glial cells, driving inflammatory signaling and disrupting homeostatic microglial functions essential for Aβ metabolism [57,204,205]. Evidence suggests that Tat and gp120 impair multiple pathways required for effective microglial Aβ clearance [206]. Tat has been shown to directly interact with APP, facilitating its localization to lipid rafts and enhancing its cleavage by β- and γ-secretases, thereby increasing Aβ production [207]. Tat colocalizes with APP in the cytosol, promoting the generation of Aβ_42_ peptides [208]. Additionally, Tat disrupts endolysosomal function, leading to the accumulation of APP, Aβ, and β-secretase within these organelles, further amplifying Aβ peptide production [207,209,210]. Despite these effects on Aβ production, impaired clearance remains the dominant driver of Aβ CNS accumulation in HIV infection. Tat also suppresses genes required for autophagosome maturation and lysosomal acidification, disrupting autophagic flux and leading to defective degradation of internalized Aβ [211]. Similarly, gp120 reduces the expression of lysosomal proton pumps and cathepsins, further diminishing degradative capacity [212,213]. These HIV proteins directly activate NF-κB and the signal transducer and activator of transcription-1 pathways, increasing transcription of pro-inflammatory cytokines such as TNF-α and IL-1β, which amplify inflammatory signaling in a feed-forward manner [214,215]. These transcriptional changes repress scavenger receptors essential for Aβ binding and uptake, including TREM2 and CD36 [216], thereby limiting both receptor-mediated Aβ internalization and phagocytic clearance.

Although Tat and gp120 are the most studied viral proteins in the context of microglial Aβ clearance, other HIV proteins may also contribute to neurodegeneration (Table 3). Nef has been shown to alter lipid raft composition and disrupt cholesterol metabolism, processes that influence APP processing and Aβ trafficking [217]. Vpr induces mitochondrial dysfunction, oxidative stress, and DNA damage responses in microglia, which can impair phagocytosis and promote inflammatory signaling [218]. Vpu interacts with host ion channels and tetherin, potentially modulating microglial membrane dynamics and cytokine release [219]. Unlike Tat and gp120, which have been directly linked to impaired Aβ clearance, the effects of Nef, Vpr, and Vpu on this process remain poorly defined. However, their capacity to disturb lipid homeostasis, mitochondrial activity, and immune signaling demonstrates that HIV-induced microglial dysfunction may be caused by multiple viral factors.

Compounding this dysfunction, chronic microglial activation in HIV is associated with inflammasome priming and increased oxidative stress, both of which disrupt endolysosomal trafficking [213,220]. NLRP3 activation, driven by HIV proteins and extracellular Aβ, leads to IL-1β release and pyroptosis, potentially amplifying glial dysfunction and neuronal injury [221,222]. HIV-related neuroinflammation also suppresses transcriptional programs linked to phagocytic competency, with a marked shift toward a disease-associated microglial phenotype, characterized by high expression of complement component 1q, ApoE, and proinflammatory cytokines, features shared with AD [223]. Notably, HIV-infected people on suppressive ART still exhibit signs of amyloid deposition, and those with HAND have shown increased cortical Aβ levels in both postmortem and neuroimaging studies [224,225]. These findings suggest that HIV may accelerate or mimic AD-like pathology via chronic disruption of microglial Aβ clearance [223].

**Table 3:** HIV proteins implicated in microglial dysfunction and possible links to Aβ metabolism.

HIV Protein	Reported Microglial Effects	Potential Impact on Aβ Handling	References
Tat	Alters receptor expression (TREM2, LRP1, SCARB1), impairs lysosomes, increases inflammatory cytokines	Reduced phagocytosis, enhanced extracellular Aβ	[207,209,210]
gp120	Activates CXCR4/CCR5, induces Ca^2+^ influx and excitotoxicity, increases BACE1 expression	Promotes amyloidogenic APP cleavage, neurotoxicity	[212,213]
Nef	Alters lipid rafts, disrupts cholesterol trafficking, modulates exosome release	May influence APP processing and Aβ aggregation	[217,226,227,228,229]
Vpr	Causes mitochondrial dysfunction, oxidative stress, cell-cycle arrest in glia	Energy deficits impair phagocytosis, promote inflammation	[230,231]
Vpu	Modulates ion channels and tetherin, alters membrane trafficking	Potential effects on cytokine release and receptor dynamics	[219]

Abbreviations: Tat: trans-activator of transcription; Gp120: glycoprotein 120; Vpr: viral protein R; Vpu: viral protein U; Nef: negative regulatory factor; TREM2: triggering receptor expressed on myeloid cells 2; LRP1: low-density lipoprotein receptor–related protein 1; SCARB1: scavenger receptor Class B Type 1; CXCR4/CCR5: C-X-C chemokine receptor 4/C-C chemokine receptor 5; BACE1: β-site APP-cleaving enzyme 1.

## 5. HIV-Associated Disruption of Microglial Aβ Clearance

### 5.1. Microglial Aβ Binding, Uptake, and Phagocytosis

**Figure 1:**
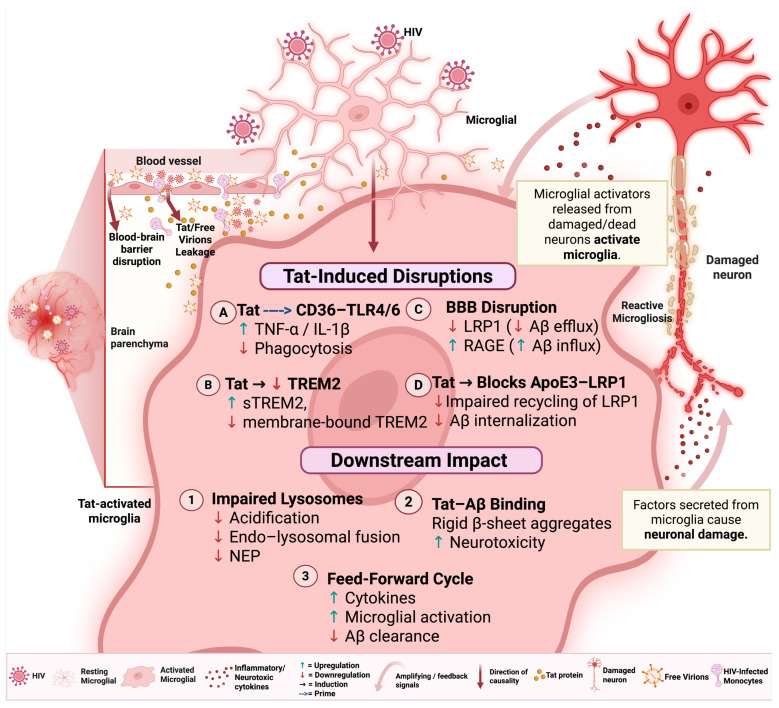
Schematic illustration of HIV Tat-induced disruption of microglial function, impairment of Aβ clearance, and neurodegeneration. Disruption of the blood–brain barrier (BBB) allows HIV-1 Tat, free virions, and/or infected leukocytes to infiltrate the brain parenchyma (upper left panel). Tat then activates resting microglia, promoting a phenotypic transformation from a homeostatic, ramified phenotype to an activated amoeboid phenotype [233]. This transition involves several receptor-mediated changes: (A) Tat primes CD36-associated inflammatory signaling, leading to increased ROS, TNF-α, and IL-1β production while paradoxically impairing CD36-mediated Aβ phagocytosis [169,232]. (B) Tat induces downregulation of TREM2, and this is associated with increased cleavage and release of soluble TREM2. (C) Tat blocks ApoE3–LRP1 interactions, impairing LRP1 recycling and reducing Aβ internalization [232]. (D) Tat-mediated BBB disruption leads to decreased LRP1-mediated Aβ efflux and increased RAGE-dependent Aβ influx [234,235,236]. The downstream impact of these changes include: (1) impaired lysosomal degradation due to reduced acidification, disrupted endo-lysosomal fusion, and loss of neprilysin [237,238]; (2) direct Tat-Aβ binding, which enhances β-sheet aggregation and Aβ neurotoxicity, thereby compounding clearance deficits [239]; and (3) a chronic feed-forward cycle of proinflammatory cytokine secretion, microglial activation, and further Aβ accumulation [214,215]. Activated microglia release neurotoxic factors (cytokines), which damage neurons and promote reactive microgliosis [221,222]. In turn, damaged or dying neurons release endogenous danger signals that further activate microglia [86,240], establishing a vicious cycle of neuroinflammation and neurodegeneration. Created in BioRender. Pociot, F. (2025) https://BioRender.com/7kobfwm.

Microglia maintain brain homeostasis by clearing Aβ through receptor-mediated binding, uptake, and phagocytosis [13]. Among the key receptors involved, CD36 recognizes Aβ fibrils and initiates their internalization and delivery to endolysosomal compartments for degradation [172]. In the context of HIV infection, viral proteins such as Tat and gp120 sustain chronic microglial activation, creating a primed inflammatory state that shifts CD36 signaling away from efficient Aβ clearance and toward excessive cytokine production and neuroinflammation (Figure 1) [169]. While CD36 is essential for Aβ recognition and clearance [169,172], its function can be subverted under inflammatory conditions. In the context of HIV infection, viral proteins such as Tat and gp120 promote chronic microglial activation and cytokine release [232], which may impair CD36-mediated clearance and contribute to a feed-forward cycle of Aβ accumulation.

HIV-mediated disruption of receptor-mediated Aβ uptake is further compounded by its effects on phagocytic pathways. Tat hampers microglial uptake and phagocytosis of Aβ, contributing to Aβ accumulation in the extracellular space [232]. This Tat-induced disruption of microglial Aβ phagocytosis involved the activation of signal transducer and activator of transcription-1 signaling and microglial shift from a phagocytic to antigen-presenting phenotype [232]. The TREM2-CD36 axis is another critical pathway affected by HIV. TREM2 enhances microglial phagocytosis of Aβ by upregulating CD36 expression through the C/EBPα transcription factor [241]. Overexpression of TREM2 leads to increased CD36 levels and improved Aβ clearance, whereas TREM2 deficiency results in reduced CD36 expression and impaired phagocytosis [241]. Although direct evidence of HIV’s impact on TREM2 is limited, alterations in CNS TREM2 may be associated with HAND. Analysis of frontal cortex tissues from HIV-infected humans showed that compared to cognitively normal individuals, in people with mild neurocognitive disorder, TREM2 levels were increased in the soluble fraction and decreased in the membrane-enriched fraction, and these alterations correlated with elevated Aβ and TNF-α transcript levels [242]. This suggests a role for dysregulated TREM2 in driving neuroinflammatory processes in HAND. This is further supported by animal studies showing that EcoHIV-infected mice exhibit increased immature and C-terminal TREM2 fragments associated with impaired cognitive performance, suggesting that HIV disrupts normal TREM2 processing [243]. Notably, cannabinoids may modulate this pathway: cannabis use in HIV-infected people has been associated with upregulated TREM2-transcripts, while cannabidiol treatment increased TREM2 mRNA and partially reversed IL-1β–induced reductions in membrane-bound TREM2 [243].

In addition to directly altering microglial receptor pathways, Tat also disrupts Aβ uptake indirectly by interfering with ApoE3–mediated clearance (Figure 1). In fact, Tat blocks ApoE3-mediated enhancement of Aβ uptake in human microglia, likely by interfering with LRP1-mediated internalization and trafficking [232]. Beyond microglia, Tat downregulates LRP1 at the BBB while upregulating the RAGE, which facilitates Aβ influx into the CNS, thereby increasing the brain Aβ burden and further compromising clearance [234,235,236,239,244].

Other key scavenger receptors, including SCARA1 (MSR1) and SCARB1, play important roles in Aβ binding and uptake in AD [245,246]. SCARA1 mediates up to 65% of microglial Aβ uptake, and its deletion results in impaired plaque clearance and worsened cognitive function in transgenic models [155,247]. Similarly, SCARB1 binds Aβ and co-localizes with amyloid plaques [245]. Despite clear evidence for the role of SCARA1 and SCARB1 in Aβ internalization and plaque removal in AD, whether HIV infection alters their expression or function in microglia remains unknown.

### 5.2. Aβ Transport and Degradation

Beyond promoting Aβ synthesis, Tat impairs Aβ clearance by inhibiting neprilysin, a zinc-dependent membrane metalloprotease responsible for degrading bioactive peptides (Figure 1). Neprilysin is produced and expressed by microglia, neurons, and astrocytes [248]. In microglia, it can function either on the cell surface or be secreted into the extracellular space, where it degrades Aβ that has not yet been internalized [249]. Tat has been shown to inhibit neprilysin activity by 80%, resulting in increased extracellular Aβ levels [237]. Tat and gp120 also impair microglial endolysosomal function.

Interactions between Tat and Aβ extend to the formation of neurotoxic complexes. Tat binds to Aβ fibrils, inducing their aggregation into rigid, multifibrillar structures with enhanced neurotoxicity [239]. These complexes exhibit increased β-sheet content and mechanical stability, leading to greater neuronal damage compared to Aβ fibrils alone [239]. These findings have been corroborated by molecular dynamics simulations showing that Tat induces conformational changes in Aβ monomers, promoting aggregation-prone structures and stabilizing preformed fibrils [209,244].

Animal studies have provided further insights into Tat’s role in AD-like pathology. Transgenic mice expressing both APP and Tat exhibited increased Aβ deposition, tau phosphorylation, and neuronal apoptosis compared to mice expressing APP alone [250]. These findings suggest that Tat exacerbates AD-like neuropathology in vivo [250]. Although the roles of neprilysin and lysosomal trafficking have been explored, it remains unknown as to whether SCARA1- or SCARB1-mediated Aβ transport and degradation are impaired by HIV. These receptors are known to internalize Aβ into lysosomal compartments in AD, but no studies have investigated whether HIV or viral proteins interfere with this degradative pathway. Addressing this gap could expand the mechanistic landscape of HAND.

## 6. Detection of Aβ Fibrils and Pathology

In addition to mechanistic studies, sensitive approaches have been developed to monitor Aβ aggregation and clearance [251]. Thioflavin-T fluorescence assays remain the gold standard for tracking fibril growth, while complementary readouts, including electron and atomic force microscopy, circular dichroism, and Raman spectroscopy, validate structural features of the aggregates [252]. At the cellular level, assays such as pH-sensitive rhodamine dye-labeled-Aβ uptake or fluorescein isothiocyanate-labeled-Aβ uptake assays, combined with lysosomal readouts including LysoTracker and cathepsin activity, provide direct measures of microglial phagocytosis and degradation capacity [253]. These tools can be applied alongside receptor blockade or knockdown to define clearance pathways mediated by CD36, TREM2, and LRP1 [84], and extended to the less studied scavenger receptors SCARA1 and SCARB1. Clinically, measurement of cerebrospinal fluid and plasma Aβ42/40 ratios and the use of amyloid-β Positron Emission Tomography now enable longitudinal tracking of amyloid pathology and better disease prognosis [254,255], including in people with HIV [256].

## 7. Therapeutic Strategies in AD and HAND

Several therapeutic strategies are currently in clinical use or under evaluation. In AD, acetylcholinesterase inhibitors (donepezil, rivastigmine, and galantamine) and the NMDA-receptor antagonist memantine remain the mainstays of symptomatic treatment [257,258]. More recently, anti-amyloid monoclonal antibodies such as lecanemab and donanemab have shown disease-modifying benefit in early symptomatic stages, although careful monitoring for amyloid-related imaging abnormalities is required [259,260]. Non-pharmacological measures, including cognitive rehabilitation, structured exercise, and vascular risk management, further contribute to preserving cognitive function and daily living skills [261]. In contrast, there is no disease-specific therapy for HAND. Optimization of combination antiretroviral therapy remains the cornerstone of HAND management, complemented by treatment of comorbidities, psychiatric and sleep disorders, and supportive approaches such as cognitive rehabilitation and exercise [262,263,264]. Future therapeutic directions for Aβ pathology in AD and HAND could include experimental strategies aimed at restoring microglial receptor trafficking, lysosomal function, and mitochondrial homeostasis.

There are other therapeutic strategies aimed at counteracting microglial dysfunction. Activation of Piezo-type mechanosensitive ion channel component-1 by pharmacological agonists has been shown to enhance microglial clearance of Aβ peptides [265]. Peroxisome proliferator-activated receptor-γ agonists such as rosiglitazone have anti-inflammatory and antiviral benefits, modulating microglial activation toward a protective phenotype [266,267]. Epigenetic modulation via selective histone deacetylase-3 inhibition shows promise in restoring a balanced microglial gene expression and reducing neuroinflammation [268]. Additionally, targeting chemokine signaling with C–C chemokine receptor type-5 antagonists and utilizing mesenchymal stem-cell-derived extracellular vesicles offer novel neuroprotective approaches to restore microglial function and limit neurodegeneration [269,270,271,272].

## 8. Conclusions

Microglia maintain CNS homeostasis through immune surveillance and clearance of Aβ, but their dysfunction is a hallmark shared by both AD and HAND [273]. In both conditions, chronic activation alters the microglial phenotype, shifting cells from a surveillance mode to a persistently reactive and often dysfunctional state. In AD, this shift is driven primarily by aging and genetic risk factors, such as TREM2 variants, which reduce Aβ uptake and impair lysosomal degradation [274,275]. In HAND, however, microglial dysfunction arises from chronic exposure to HIV and viral proteins, persistent immune activation, and oxidative stress linked to CNS viral reservoirs [233,276]. These factors disrupt homeostatic gene expression programs and phagocytic efficiency, limiting the ability of microglia to process and degrade extracellular Aβ. Multiple studies now demonstrate that these HIV-induced microglial alterations contribute to enhanced Aβ accumulation and neuronal damage, thereby accelerating cognitive impairment and HAND.

Despite this progress, significant gaps remain. While receptors such as CD36, TREM2, and LRP1 are well studied in Aβ clearance, the roles of macrophage scavenger receptors SCARA1 and SCARB1 in HAND are not well defined. It is unclear if HIV proteins like Tat and gp120 suppress these receptors transcriptionally, mislocalize them, or impair their function. Moreover, the precise sequence connecting viral exposure, receptor dysregulation, lysosomal dysfunction, and Aβ accumulation is not fully mapped. Although lysosomal defects, mitochondrial dysfunction, and inflammasome activation are observed in both AD and HAND, the specific signaling pathways that maintain these abnormalities in HIV infection require further elucidation.

Current therapeutic approaches, including optimization of antiretroviral therapy and symptomatic management, can stabilize patients but do not directly address these Aβ clearance deficits. Promising future directions should include experimental strategies targeting receptor trafficking, lysosomal repair, and mitochondrial function. Future research should also define how, in addition to Tat and gp120, less-studied HIV proteins such as Nef, Vpr, and Vpu converge on receptor regulation and trafficking, lysosomal competence, and inflammatory signaling. Thereafter, preclinical testing of therapeutic strategies that target receptor trafficking, lysosomal repair, and mitochondrial health should help assess their potential as disease-modifying therapies against microglia-mediated Aβ pathology in AD and HAND.

## Figures and Tables

**Table 1:** Toll-like receptors regulating microglial Aβ clearance and inflammation in AD.

TLR	Aβ Role	Mechanism of Action	Human Evidence	Animal Evidence	References
TLR2	Recognizes Aβ; skews microglia to inflammatory state	Interacts with CD14; activates MyD88–NF-κB pathway; **↓** phagocytosis, ↑ proinflammatory cytokines (TNF-α, IL-1β, IL-6)	Co-localized with CD14+ microglia near plaques in human AD tissues	TLR2^–/–^ mice exhibit ↓ cytokines and ↑ Aβ clearance; anti-TLR2 antibody ↓ ROS in vitro	[142,143,145,147]
TLR4	Recognition of fibrillar Aβ triggers inflammation and clearance	Binds Aβ via CD14–MD2 co-receptor complex; activates MyD88-dependent NF-κB and MAPK pathways **↑** TNF-α, IL-1β, ROS, iNOS	Upregulated in plaque-associated microglia in AD brains	TLR4^–/–^ mice show impaired Aβ clearance, ↑ plaque burden, cognitive decline	[140,141,145]
TLR9	Peripheral modulation of Aβ burden	Endosomal TLR; activated by CpG ODNs, enhances peripheral monocyte activation and recruitment into the CNS; does not engage Aβ directly.	Not upregulated in microglia in AD brains	Systemic CpG ODN injection ↓ cortical Aβ by 66% and improves memory in AD mice	[146]

Annotations: ↓ = downregulation, ↑ = upregulation. Abbreviations: MyD88: myeloid differentiation primary response 88; TNF-α: tumor necrosis factor-alpha; IL: interleukin; ROS: reactive oxygen species; iNOS: inducible nitric oxide synthase; NF-κB: nuclear factor kappa-light-chain-enhancer of activated B cells; MAPK: mitogen-activated protein kinase; CpG ODNs: CpG oligodeoxynucleotides; CNS: central nervous system; Aβ: amyloid-beta; TLR: toll-like receptor; AD = Alzheimer’s disease.

## Abbreviations

The following abbreviations are used in this manuscript 5XFAD (Five Familial Alzheimer’s Disease Mutations mouse model); AD (Alzheimer’s Disease); AKT (Protein Kinase B); APOE (Apolipoprotein E); APP (Amyloid Precursor Protein); APPPS1 (Amyloid Precursor Protein/Presenilin 1 transgenic mouse model); ART (Antiretroviral Therapy); Aβ (Amyloid-beta); BBB (Blood–Brain Barrier); CD (Cluster of Differentiation); CNS (Central Nervous System); CpG ODN (Cytosine-phosphate-Guanosine Oligodeoxynucleotide); DAMPs (Damage-Associated Molecular Patterns); DNAX (DNAX-activating protein of 12 kDa); EOAD (Early-Onset Alzheimer’s Disease); ERK1/2 (Extracellular Signal-Regulated Kinase 1/2); gp120 (Glycoprotein 120); HAND (HIV-Associated Neurocognitive Disorders); HO-1 (Heme Oxygenase-1); IL (Interleukin); iNOS (Inducible Nitric Oxide Synthase); JNK (c-Jun N-terminal Kinase); LOAD (Late-Onset Alzheimer’s Disease); LRP1 (Low-Density Lipoprotein Receptor-Related Protein 1); MAPK (Mitogen-Activated Protein Kinase); MD-2 (Myeloid Differentiation factor 2); MSR1 (Macrophage Scavenger Receptor 1); mTOR (Mechanistic Target of Rapamycin); mTORC1 (mTOR Complex 1); MyD88 (Myeloid Differentiation Primary Response 88); NF-κB (Nuclear Factor Kappa-light-chain-enhancer of Activated B cells); NFTs (Neurofibrillary Tangles); NLRP3 (NOD-, LRR- and pyrin domain-containing protein 3); NO (Nitric Oxide); PAMPs (Pathogen-Associated Molecular Patterns); PI3K (Phosphoinositide 3-Kinase); PRRs (Pattern Recognition Receptors); PS2APP (Presenilin 2 Amyloid Precursor Protein mouse model); Vpu (Viral protein U); Vpr (Viral Protein R); Nef (Negative Regulatory Factor); RAGE (Receptor for Advanced Glycation End Products); ROS (Reactive Oxygen Species); SCARA1 (Scavenger Receptor Class A Member 1); SCARB1 (Scavenger Receptor Class B Member 1); SCARB2 (Scavenger Receptor Class B Member 2); SR-AI/II (Scavenger Receptor Class A Member I/II); SR-BI (Scavenger Receptor Class B Type I); Tat (Trans-Activator of Transcription); TLR (Toll-Like Receptor); TNF-α (Tumor Necrosis Factor-alpha); TREM2 (Triggering Receptor Expressed on Myeloid Cells 2); TSC1 (Tuberous Sclerosis Complex 1).
